# Alveolar bone remodeling in virtually planned, bone-grafted vs non-grafted guided flapless implant surgery in the anterior maxilla: a cross-sectional retrospective follow-up study

**DOI:** 10.1007/s10006-022-01048-z

**Published:** 2022-02-17

**Authors:** Fredrik Åkesson, Liene Zamure-Damberga, Stefan Lundgren, Mats Sjöström

**Affiliations:** 1 Oral and Maxillofacial Surgery, Department of Odontology, Faculty of Medicine, Umeå University, 90185 Umeå, Sweden; 2 Department of Conservative Dentistry and Oral Health, Faculty of Dentistry, Riga Stradins University, Riga, Latvia

**Keywords:** Alveolar bone grafting, Virtual implant planning, Endosseous implants, Flapless guided surgery, Buccal bone loss

## Abstract

**Purpose:**

In patients who underwent virtual planning and guided flapless implant surgery for teeth missing in the anterior maxilla, we compared buccal bone loss between those treated with and without autogenous bone augmentation.

**Methods:**

Of 22 patients with teeth missing because of trauma or aplasia, 10 (18 implant sites) were reconstructed with buccally placed bone graft harvested from the mandibular ramus, and 12 were non-reconstructed (16 sites). Baseline cone-beam computed tomography allowed for implant planning using the NobelClinician® software and was performed again at 1 year after functional loading. The marginal bone level was assessed radiographically at post-implant baseline and at follow-up.

**Results:**

At follow-up, buccal bone loss differed significantly between groups at the central level of the implant (*p* = 0.0005) but not at the coronal level (*p* = 0.329). The mean marginal bone level change was 0.6 mm, with no significant between-group difference (*p* = 0.876). The actual implant position often deviated in the vertical or sagittal plane by an average of 0.3–0.6 mm from the planned position.

**Conclusion:**

Compared with non-reconstructed patients, reconstructed patients experienced significantly more buccal bone loss at the central level of implants. The groups did not differ at the coronal level or in marginal bone loss, possibly because of the more augmented bone at the central level among reconstructed patients. Differences between planned versus actual implant positions should be considered in situations of limited bone volume at the planned implant site.

## Introduction

Restoring missing teeth with single implants in the esthetic zone of the anterior maxilla is a demanding treatment. If a tooth is missing because of trauma or aplasia, often the alveolar bone must be restored in addition to the tooth for hard and soft tissue support and support of the upper lip. If the pre-operative clinical and radiographic examination indicates insufficient alveolar process bone volume for tissue support and implant placement, the first step is pre-implant bone reconstruction. Many techniques have been described, including locally harvested autogenous bone grafts [1] with or without use of membranes [2], autogenous bone grafts combined with xenografts [3], synthetic materials and their combinations with antibiotics [4], or allografts [5], and bone distraction techniques [6]. Each of these approaches has shown good, predictable results, but outcomes are technique sensitive. In patients with a healed extraction socket and subsequent hard and soft tissue atrophy, an autogenous bone graft placed prior to implant surgery is extensively documented in the literature [1, 2, 7]. One important factor for a predictable result of local alveolar bone grafting is the degree of postoperative graft volume change that takes place during initial healing and remodeling. Several studies on larger grafts in edentulous jaws have reported long-term post-grafting volume reduction of up to one-third of the original bone volume [8, 9]. Low invasive surgical procedures should be the method of choice for preserving alveolar bone volume. Flapless surgery for implant placement has been reported to reduce peri-implant bone loss compared to implant placement with flap preparation [10], but no significant differences have been reported [11] for implant survival, changes in marginal bone level, or complications.

One review of guided surgery [12] included four studies using the NobelGuide® system. The review authors concluded that implant survival with guided surgery was comparable to the estimated overall survival rate (95.6% during 5 years), despite the complex nature of the treatments performed with guided surgery. Implant placement for a single tooth in the anterior maxilla is challenging because of limited interdental space and bone volume. D’haese et al. (2017) discussed the importance of accuracy in critical anatomical situations and concluded that knowledge of implant deviation from the planned implant placement is important when using guided surgery [13]. Indeed, the planned implant position always differs from the actual position [14], and each implant site must be measured individually to track actual bone level changes. Few published studies have described bone loss and the success rate of single implants for replacing teeth lost because of, e.g., trauma or aplasia.

To address these gaps, the aim of this controlled, quality-of-care, 1-year follow-up study was to investigate buccal and marginal bone remodeling after reconstruction with buccal autologous bone grafting in the anterior maxilla followed by virtually planned guided implant placement with flapless surgery. We compared outcomes with this approach in patients with missing teeth because of trauma or aplasia to results with patients without reconstruction but with flapless guided surgery in healed alveolar processes. Our null hypothesis was that there would be no difference between remodeling for a reconstructed alveolar process compared to a non-reconstructed alveolar process.

## Materials and methods

### Patients

The present retrospective patient radiological study included 22 patients with 34 implants (13 men and 9 women) referred to the Department of Oral and Maxillofacial Surgery, Umeå University Hospital, for implant treatment during 2014–2017. The inclusion criterion for selection of patients to the study was loss of one to three teeth in the anterior maxilla due to trauma or tooth aplasia and treated with virtual planning and guided implant surgery with or without the need for pre-implant alveolar bone grafting. Table 1 summarizes age, sex, cause of tooth loss, and implant sites for the included patients. None of the participants suffered from medical conditions that could have affected dental implant survival. All patients were non-smokers. The study followed the ethical principles of the World Medical Association Declaration of Helsinki 2013 [15], and informed consent was obtained from all participants included in the study.

**Table 1:** Baseline comparisons between reconstructed and non-reconstructed groups treated with implants, with the surgical procedure performed using the NobelGuide system

Parameter	Reconstructed patients (*n* = 10)	Non-reconstructed patients (*n* = 12)	*p*
Age in years, mean (± *SD*)	33 (12)	32 (13)	0.88*
Sex (f/m), *N* = 13/9	7/3	6/6	0.41**
Cause of tooth loss			0.64**
Aplasia (11 sites)	4	7	
Trauma (23 sites)	14	9	
Implant sites (*n*)			
Total	18	16	0.41**°
Central incisors (1)	10	5	
Lateral incisors (2)	5	7	
Canines (3)	3	4	
Average time from baseline CBCT (after reconstruction) to minimum 1 year after loading implants, months (± *SD*)	19.4 (3.1)	25.9 (6.2)	0.01*
Average time between baseline CBCT and implant placement, months (± *SD*)	1.3 (0.7)	5.7 (3.9)	< 0.01*
Average time between implant placement and CBCT minimum 1 year after loading implants, months (± *SD*)	18.1 (3.2)	20.2 (3.5)	0.18*

*SD* standard deviation

^*^Two-sample *t*-test

^**^Fisher’s exact test

°Omnibus p value of fixed effects for central measure from the mixed model. The effects for occasion (baseline and follow-up) and reconstruction were calculated in reference to baseline and no reconstruction, respectively. The effects for site were calculated using the central incisor as reference

### Reconstructed patients

In 10 patients with missing teeth, 18 sites had significant bucco-palatal atrophy, and the bone volume was insufficient for implant placement. These patients were treated with a bone block graft harvested from the mandibular ramus 6 months prior to implant placement. Two of the ten patients had missing teeth because of aplasia in four sites, and the remaining eight patients had 14 teeth missing in total because of trauma.

### Non-reconstructed patients (controls)

In 12 patients with missing teeth, 16 sites had sufficient bone volume for implant placement. Four patients had missing teeth in seven sites because of aplasia, and eight patients had nine teeth missing in total because of trauma.

### Implant planning procedure

Prior to the virtual planning procedure, all patients were examined with cone-beam computed tomography (CBCT). Patients with insufficient bone volume for implant placement were scheduled for bone augmentation, and at 6 months after bone grafting, a new CBCT examination was performed. The post-augmentation CBCT for the reconstructed group and the pre-treatment examination for the non-reconstructed group were considered to be the baseline CBCT examination. The NobelClinician® (Nobel Biocare Guided Surgery Center, Mechelen, Belgium) software was used for treatment planning and template production. All patients except for the first three were planned with Smart Fusion® (Nobel Biocare, Kloten, Switzerland), in which intra-oral 3D scanning was combined with CBCT data. In the first three patients, a separate radiological guide was used for data fusion. Immediately after implant placement, an intra-oral radiographic examination of the marginal bone using a parallel technique was performed on all participants for baseline assessment of each implant’s marginal bone level.

### Bone grafting procedure

All patients were premedicated with 2 g of amoxicillin, and both surgical sites were prepared with local anesthesia (20 mg/ml lidocaine, 12.5 µg/ml adrenaline ASTRA AB). At the donor site, after a mucoperiosteal flap was raised, the cortical bone graft was outlined with a piezotome on the lateral border of the retromolar area and ascending mandibular ramus and then harvested with an osteotome. At the recipient site, a trapezoid mucoperiosteal flap was raised, and the donor graft was tailored to fit the recipient site. Sharp edges and margins were trimmed to avoid risk of tearing the overlying mucosa. The bone was fixed with a single screw (KLS Martin® screw, 2-mm diameter, 9–12 mm in length depending on bone block thickness). Both donor site and recipient site flaps were closed, so that the length of the flap in the recipient site was increased with a reversed periosteal incision. Six months later, all patients returned for CBCT and clinical examinations. Virtual 3D planning was performed, and a surgical guide was fabricated from the CBCT and scanning data.

### Implant placement procedure

In all 34 implant sites, NobelActive® (Nobel Biocare, Karlskoga, Sweden) dental implants were placed with a flapless surgical technique. The implant placement procedure was performed using the teeth-supported fully guided template for drilling and implant placement in both groups [16]. For maximum stability, templates were extended bilaterally to the first molar. The implants were loaded 6–8 weeks after placement with a screw-retained supra-construction. After a minimum of 1 year of loading, all 22 participants were examined with a follow-up CBCT for comparison with baseline bone and by intra-oral radiography for comparing the marginal bone level with baseline. The implant survival rate was calculated after a minimum of 1 year of functional loading.

### Radiological evaluation

**Fig. 1:**
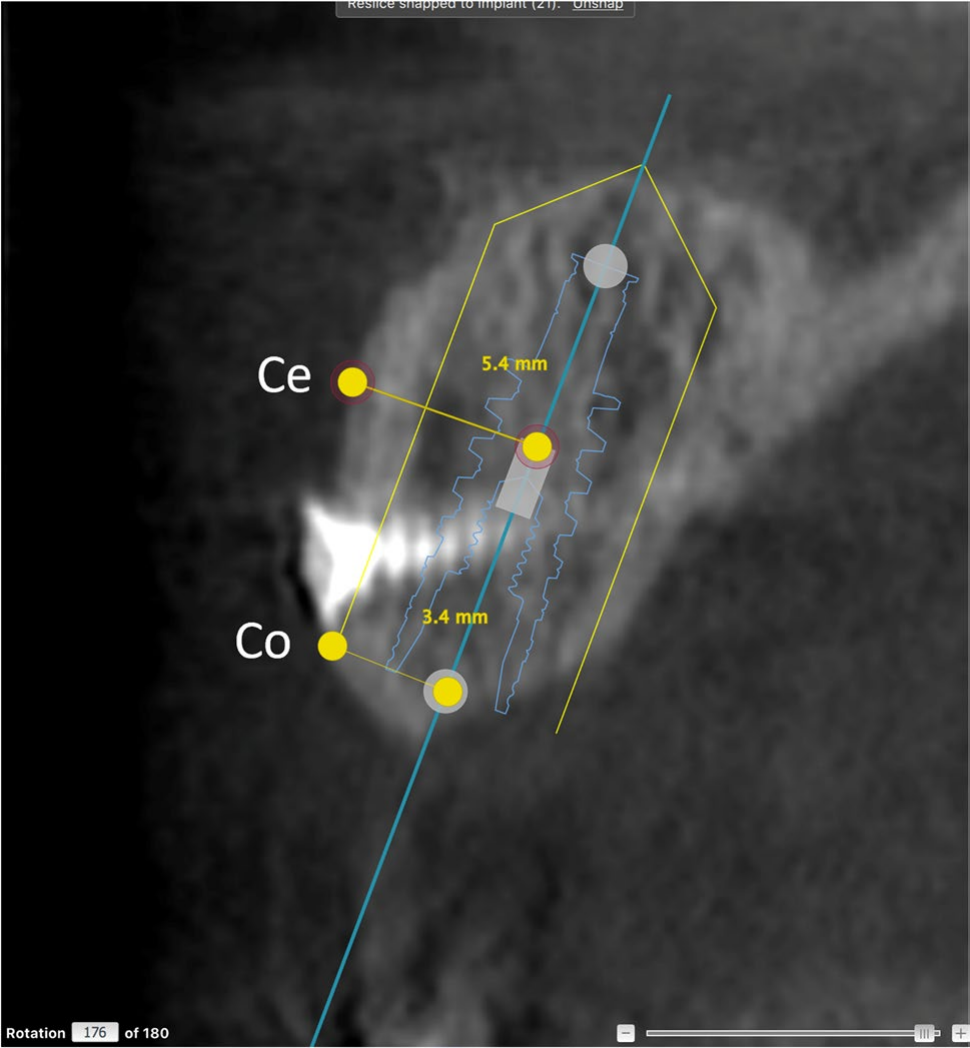
CBCT examination showing baseline planning of implant placement using NobelClinician® software in a reconstructed patient. Measures of baseline buccal bone were performed at the central (Ce) and coronal (Co) levels of the guide for implant placement

**Fig. 2:**
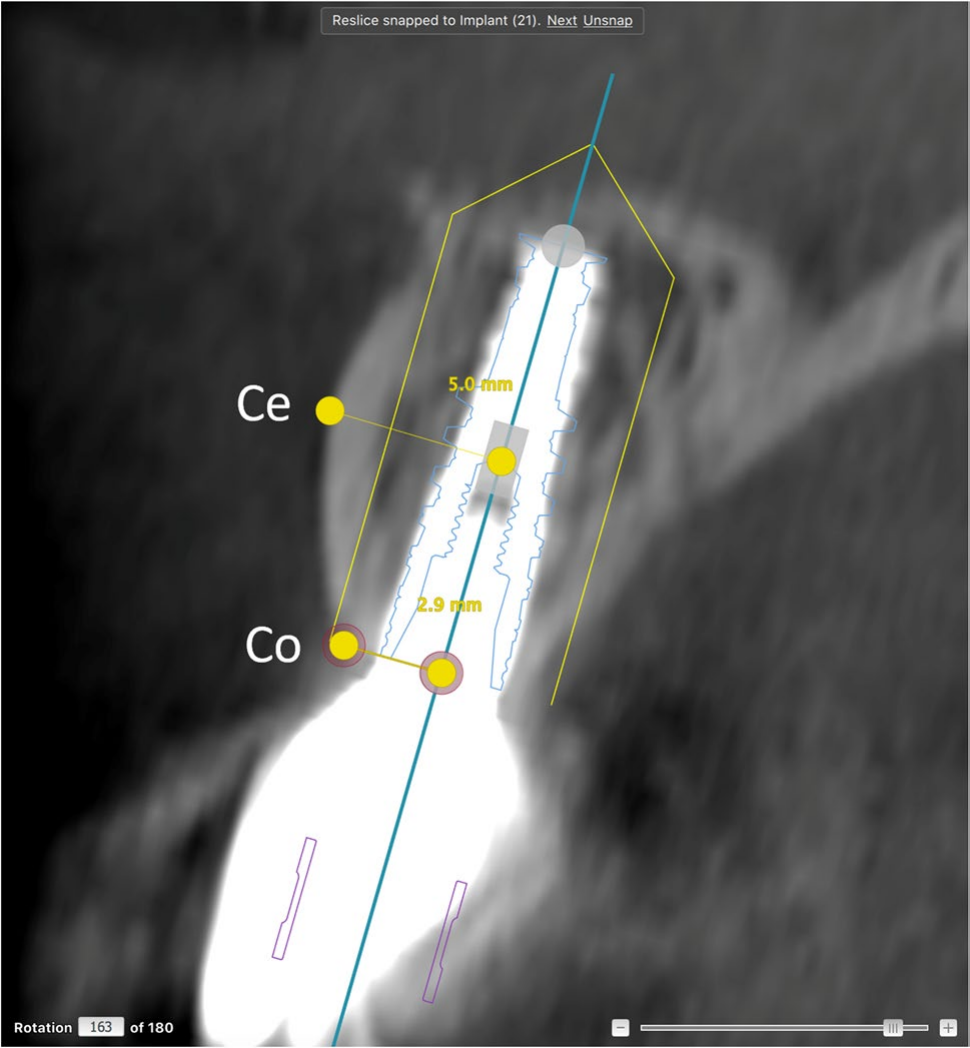
Follow-up CBCT examination in a reconstructed patient. The NobelClinician® software planning instrument was superimposed on the actual placement of the implant. Measures of buccal bone were performed at the central (Ce) and coronal (Co) levels of the implant, taking differences between the actual and the planned implant placement into consideration. In this case, the planned and the actual placement corresponded to one another

The CBCT (3D Accuitomo 170; J Morita MFG Corporation, Kyoto, Japan) examinations were performed with the following settings: at baseline, volume 5 × 10 cm, 85 kV, 7 mA, and 360° for NobelGuide planning, and at the minimum 1-year follow-up, volume 4 × 4 cm or 6 × 6 cm (depending on the number of implants), 85 kV, 7 mA, and 360° (Figs. 1 and 2).

**Fig. 3:**
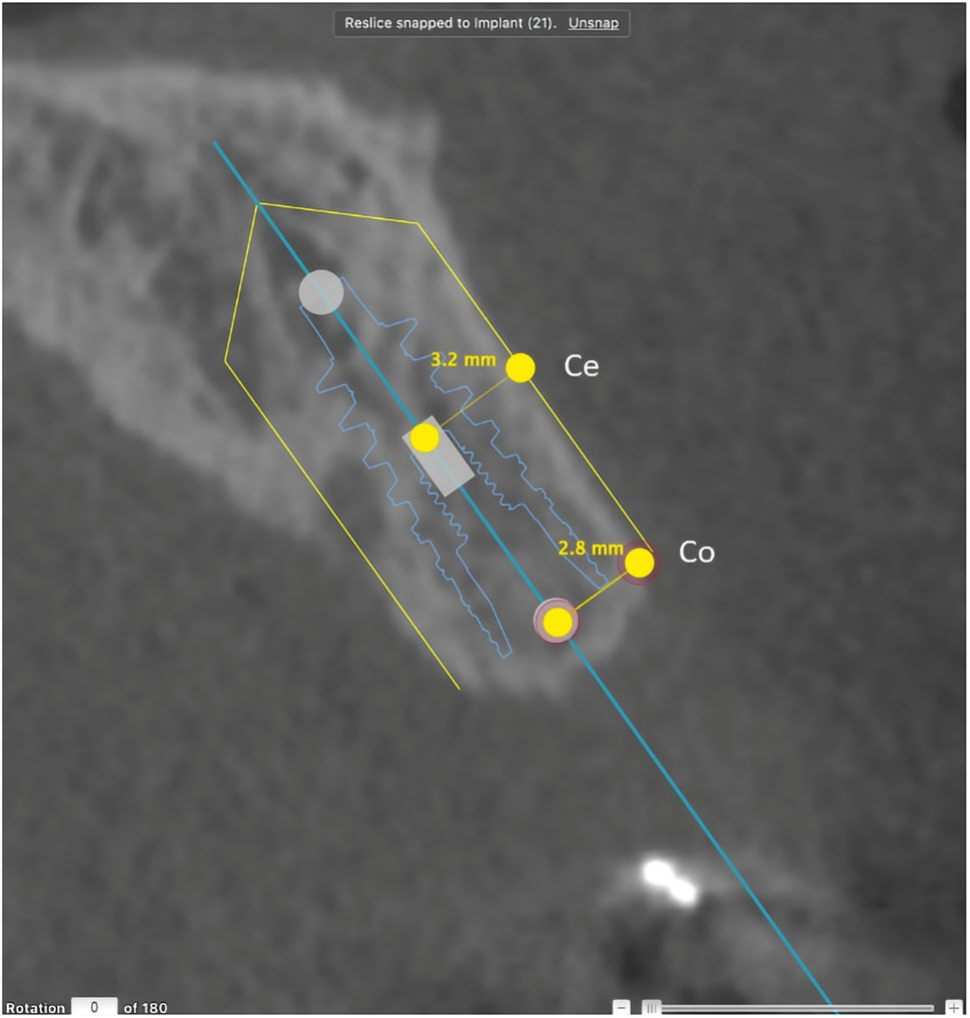
CBCT examination showing baseline planning of implant placement using NobelClinician® software in a non-reconstructed patient. Measures of baseline buccal bone were performed at the central (Ce) and coronal (Co) levels of the guide for implant placement

**Fig. 4:**
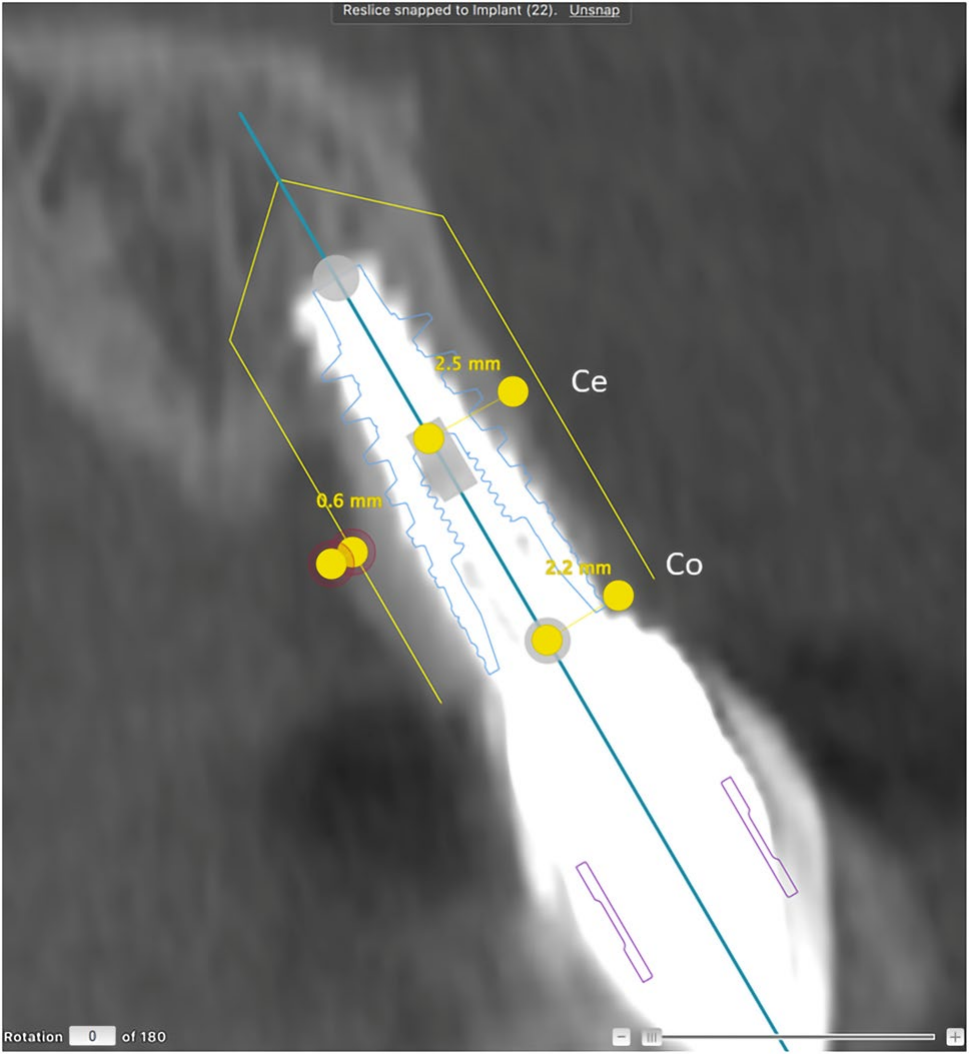
Follow-up CBCT examination in a non-reconstructed patient. The NobelClinician® software planning instrument was superimposed on the actual placement of the implant. Measures of buccal bone were performed at the central (Ce) and coronal (Co) levels of the implant, taking differences between the actual and the planned implant placement into consideration. In this case, there was a sagittal deviation of 0.6 mm between the planned and the actual placement

Two of the authors (F.Å. and L.Z.) performed the assessments of the radiographic examinations after the initial calibration. They made the assessments twice, independent of each other, and inter- and intra-rater reliability values were calculated. Baseline measurements were performed on the pre-operative CBCT examinations using the planned placement of the implants in the NobelClinician® software. The amount of buccal bone was measured perpendicular from the central long axis of the implant planning instrument (superimposed at follow-up) to the buccal surface of the alveolar process. Measurements included buccally grafted bone when present and were made at the central and coronal levels of each implant (Figs. 1 and 3). Follow-up CBCT examinations were assessed using the NobelClinician® software (Nobel Biocare Guided Surgery Center, Mechelen, Belgium), superimposing the planning instrument on the implant, measuring at the same levels as at baseline (Figs. 2 and 4).

We assessed the accuracy of the actual position of the implant compared to the pre-operative digitally planned position (Fig. 3). An optimal section in relation to the long axis of the implant first was chosen according to anatomy. Then, measurement of the buccal bone thickness was adjusted for differences in depth placement and sagittal plane placement and compared to the baseline planning (Figs. 2 and 4). In case of disagreement in measures, consensus was reached, and consensus results were used for statistical analyses of changes in buccal bone thickness among patients with and without buccal bone augmentation.

**Fig. 5:**
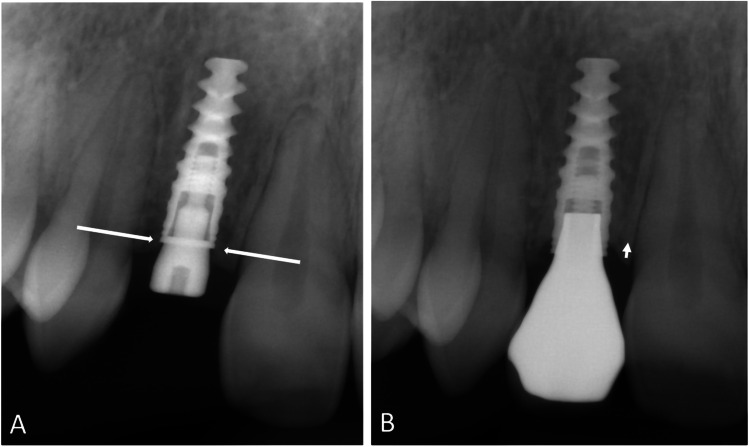
Measurement of marginal bone level related to the level of the implant shoulder mesial and distal to the implant (marked with arrows) on intra-oral radiographs exposed perpendicular to the implant shoulder. **A** Examination at baseline; the bone level exceeded the platform of the implant and were scored as 0. **B** Examination after 1 year of loading

Changes in marginal bone level were measured mesially and distally to the implant on the intra-oral radiographs exposed perpendicular to the implant shoulder, before and 1 year after implantation. The distance between the implant platform and first bone–implant contact was measured. Cases in which the bone level exceeded the platform of the implant were scored as 0 (no bone loss) (Fig. 5 A and B).

### Statistics

The relationship between buccal bone loss and reconstruction versus non-reconstruction was modeled using a linear mixed effect analysis. We used reconstruction, time, and implantation site as fixed effects and a random intercept for subjects. *p* values were calculated using the likelihood ratio test with Satterthwaite’s method for approximating degrees of freedom. Analyses were performed with R (R v4.0.3, R Core Team) and lme4 (lme4 v 1.1–17 [17]).

The intra-class correlation (*ICC*) using the single random raters (*ICC*2) was calculated for determining intra-rater and inter-rater reliability. Calculations for inter-rater reliability were based on the initial assessment of buccal bone thickness and marginal bone level, and those for intra-rater reliability were based on the first and second assessments. These analyses were conducted in R (R v4.0.3, R Core Team), and figures were produced using the package ggplot2 (Wickham, 2016 [18]). The linear mixed effect models were fitted with lme4 [17], and the *ICC* was calculated with psy (Falissard, 2012 [19]).

For descriptive statistics, we performed group comparisons by sex with a two-sample test for equality of proportions with continuity correction of the *p* value. The two-sample *t*-test was used for comparisons between groups for time between baseline and implant placement, implant placement and follow-up, baseline and follow-up, and age.

## Results

### Differences between planned and final implant placement

The actual placement in depth (vertical plane) and coronal and central bucco-palatal plane (sagittal plane) of the implants compared with planned placement using a surgical guide deviated by a mean 0.3–0.6 mm, with no statistically significant difference between groups (*p* = 0.49, *p* = 0.99, *p* = 0.75; Table 2). All implants were functional at follow-up, for an implant survival rate of 100% for both groups.

**Table 2:** Average deviation (± standard deviation) between virtual implant planning and actual placement, mean value expressed in millimeters, for reconstructed and non-reconstructed implant sites with surgical implant placement performed with the NobelGuide system

Parameter	Reconstructed patients (*n* = 10)	Non-reconstructed controls (*n* = 12)	*p*
Depth deviation (vertical)	0.46 (0.58)	0.29 (0.75)	0.49*
Bucco-palatal deviation (sagittal)			
At the coronal level of the implant	0.48 (0.39)	0.48 (0.47)	0.99*
At the central level of the implant	0.52 (0.45)	0.58 (0.68)	0.75*

^*^Two-sample *t*-test

### Buccal bone loss

**Fig. 6:**
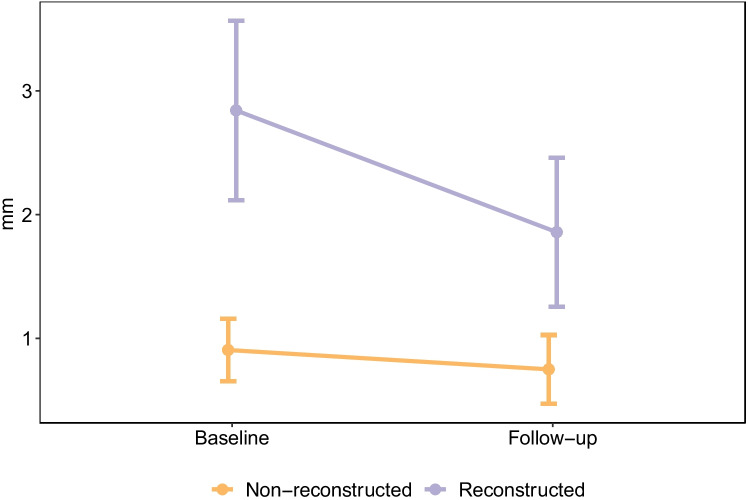
Buccal bone loss at the central level. The implants placed in buccally reconstructed alveolar bone showed significantly (*p* < 0.001) more bone loss compared to implants placed in non-reconstructed alveolar bone at the 1-year follow-up

**Fig. 7:**
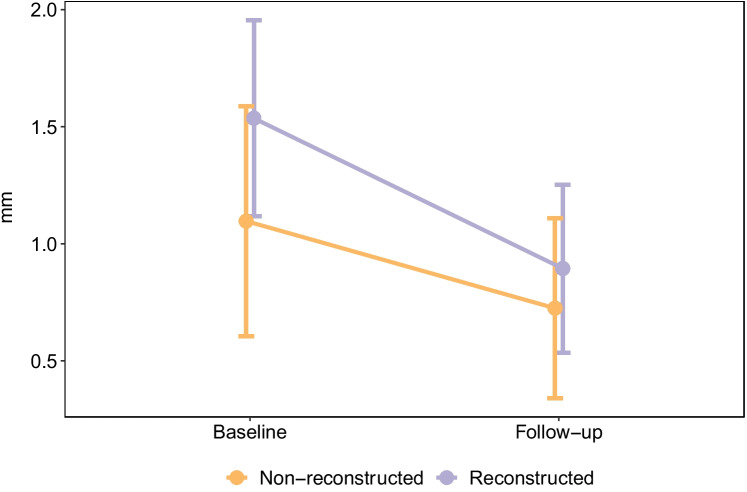
Buccal bone loss at the coronal level in implants placed in reconstructed alveolar bone compared to implants placed in non-reconstructed alveolar bone at 1-year follow-up (*p* = 0.329)

The differences in buccal bone loss between groups at baseline compared to follow-up were analyzed and adjusted for the mixed models at the central and coronal levels of the implants. Among reconstructed patients and when compared to bone thickness at baseline (prior to implant placement), there was significant buccal bone loss at the 1-year follow-up at the central level (− 0.61 mm, 95% confidence interval (*CI*): − 1.02 to − 0.20, *p* = 0.005) (Fig. 6) and the coronal level (− 0.53 mm, 95% *CI*: − 0.81 to − 0.25, *p* = 0.000) (Fig. 7). The estimated effect on buccal bone loss for implants placed in reconstructed alveolar bone at the central level was significant (1.44 mm; 95% *CI*: 0.75 to 2.2, *p* = 0.0005) compared to non-reconstructed patients (Fig. 6). The difference in buccal bone loss at the coronal level was non-significant (*p* = 0.329) for implants placed in alveolar bone with buccal bone augmentation compared to the non-reconstructed patients (Fig. 7). The position of the implants had no impact on buccal bone loss.

### Marginal bone level

**Fig. 8:**
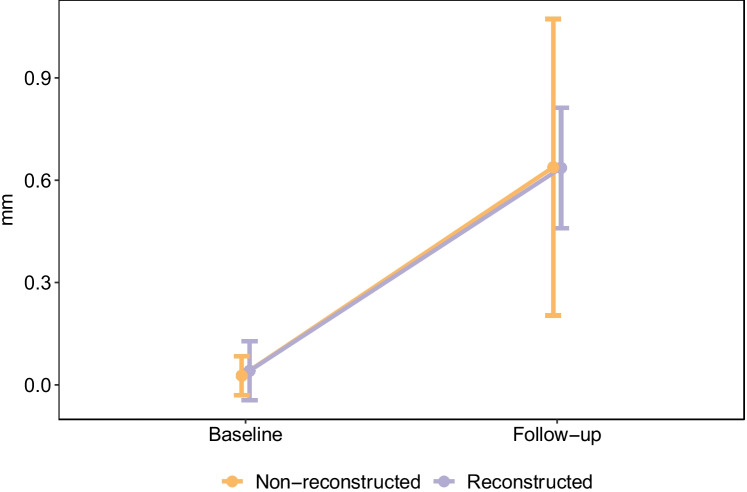
Marginal bone loss after a minimum of 1 year of loading the implant, between reconstructed and non-reconstructed implant sites. The bone loss was significant between baseline and follow-up in both groups, but with no significant difference between groups (*p* = 0.876)

The average change in marginal bone level was 0.60 mm, and marginal bone loss did not differ for implants at reconstructed versus non-reconstructed sites (Fig. 6)*.* Both groups showed bone loss between baseline and follow-up, but without significant between-group differences (*p* = 0.876; Fig. 8). Implant position in the anterior maxilla had no effect on marginal bone loss.

### Intra- and inter-rater reliability

The intra-rater reliability for the two observers for the first and second assessments was calculated, yielding *ICC* values of 0.98 (95% *CI*: 0.97 to 0.98) for assessment of buccal bone thickness and 0.91 (95% *CI*: 0.85 to 0.94) for the marginal bone level. The inter-rater reliability was also determined, yielding *ICC* values of 0.96 (95% *CI*: 0.93 to 0.98) for assessment of buccal bone thickness and 0.89 (95% *CI*: 0.79 to 0.94) for the marginal bone level.

## Discussion

The present results indicate that buccal bone loss is more pronounced at the central level of the implant in patients reconstructed with buccal bone augmentation compared to that in non-reconstructed patients. However, the two patient groups did not differ at the coronal level, possibly because the bone thickness was overcorrected at grafting and thus higher in the central portion of the graft (Fig. 6).

Zuiderveld et al. [20] used a similar technique to investigate buccal bone thickness with implants immediately placed in fresh extraction sockets and simultaneously augmented within the socket between the buccal bone wall and the implant. Their test group further received a connective tissue graft placed outside of the socket in a buccal supra-periosteal envelope flap. In their patients, the average buccal bone loss was − 0.84 mm in the test group and − 0.46 mm in the control group. In the present study, marginal bone loss between the grafted and non-grafted implants site did not differ significantly, which corresponds to the results from Zuiderveld et al. [20].

According to criteria for implant success, survival, or failure described by Misch et al. [21], the implant cohort in our study would be classified as having had a 100% success rate. Of note, the follow-up time was only 1 year, and only 34 implants were placed.

The mandibular ramus is a well-established area for harvesting autogenous bone, with low morbidity [1] and easy access [22]. A healing period of 3 to 6 months is reported for optimal healing prior to implant placement [23]. In the present study, the healing period for the bone graft was 6 months prior to implant placement for all patients.

We found that in some patients, the implant position after placement differed somewhat from the planned implant position (Fig. 4), with an average deviation in the vertical or sagittal plane of 0.3–0.6 mm. The reconstructed and non-reconstructed groups did not differ in positional deviations, and the values obtained in this study correspond well to earlier reports [24, 25].

Vercruyssen et al. [26] reported on guided surgery in edentulous patients, using mucosal support and bone anchor pins for template stabilization and found that the vertical plane (depth) was the main source of inaccuracy. In the present study, we used tooth-supported templates, which are considered to be more stable. Nevertheless, minor movements in a tooth-supported guide are always possible during the surgical procedure and can result in a discrepancy from the virtually planned implant position. The sleeve in the guide and the drill and fixture carrier have loose fittings that also might contribute to changes in implant direction in alveolar bone, and force on the drill can contribute to a change in depth.

One advantage with flapless surgery using a guided technique is that it achieves higher precision and accuracy in implant positioning. Thus, it is a suitable choice especially for complex procedures and conditions in the aesthetic zone with limited pre-existing bone volumes and limited interdental available space [27]. The technique with virtual planning after CBCT examinations, prior to guided surgery, allows for analysis of implant placement precision and changes in the volume of alveolar bone supporting the implant. In the pre-operative planning of implant placement in the esthetic zone, the thickness of the buccal bone plate is important. In cases with limited bone, using a bone graft is intended to improve conditions for implant placement. The current results indicate that buccal bone grafts decrease more compared to non-grafted situations at the central level of the implant but not at the coronal level. In an earlier study, the buccal bone plate changed to the same extent, regardless of the thickness, and implants placed with virtual planning and flapless surgery resulted in less buccal bone resorption compared to implants placed with flaps [28]. The authors raised the question of the importance of a thick buccal bone plate for implant success. Younes et al. [29] analyzed implant position among implants placed freehand, by pilot-drill guidance, and under full guidance and concluded that fully guided implant placement should be considered in cases of limited bone volumes between neighboring teeth. However, as in the present study, these authors also concluded that the actual implant placement sometimes deviates from the virtual planning.

A strength of this study is that all patients were examined with the same radiographic technique at the same clinic. Different surgeons performed the implant placement. All radiographic assessments were performed individually by two calibrated and experienced observers, and the results were based on their consensus. The decision regarding reconstruction depended on the individual surgeon’s experience. Virtual planning gives good information about the available bone volume, resulting in a lower risk for bias in the treatment decision. Blinding the radiographic examinations was not possible because the implant was placed in the alveolar process, indicating the sequence in the treatment. Limitations of the study were that the sample size was small and the follow-up time was only 1 year. Further information from long-term follow-up of the patients would be of interest.

The analyses of buccal bone loss after alveolar bone reconstruction and implant placement were based on CBCT data from examinations at follow-up. In routine clinical practice, follow-up with intra-oral radiographic examination of the implant site is common, but assessment of the buccal bone thickness is not possible without a CBCT examination.

## Conclusion

In conclusion, the amount of buccal bone loss was higher at the central level of implants in patients treated with autogenous bone augmentation in the anterior maxilla compared to patients without bone augmentation. Therefore, our null hypothesis was partly rejected. Both reconstructed and non-reconstructed patients had implants placed after virtual planning and guided flapless implant surgery using the NobelClinician software. The two groups did not differ in bone loss at the coronal level of the implant or in marginal bone loss. The actual position of the implant placement compared to the virtually planned position often differed in the sagittal or vertical dimension, which should be considered when the available alveolar bone for the implant is severely limited.
